# Non-Canonical Functions of Adenosine Receptors: Emerging Roles in Metabolism, Immunometabolism, and Epigenetic Regulation

**DOI:** 10.3390/ijms26157241

**Published:** 2025-07-26

**Authors:** Giovanni Pallio, Federica Mannino

**Affiliations:** 1 Department of Biomedical and Dental Sciences and Morphological and Functional Imaging, University of Messina, Via C. Valeria, 98125 Messina, Italy; gpallio@unime.it; 2 Department of Medicine and Surgery, University of Enna “Kore”, Contrada Santa Panasia, 94100 Enna, Italy

**Keywords:** adenosine receptors, metabolism, immunometabolism, epigenetic regulation, cancer immunotherapy, inflammation

## Abstract

Adenosine receptors (ARs) are G protein-coupled receptors that are widely expressed across tissues, traditionally associated with cardiovascular, neurological, and immune regulation. Recent studies, however, have highlighted their non-canonical functions, revealing critical roles in metabolism, immunometabolism, and epigenetic regulation. AR subtypes, particularly A2A and A2B, modulate glucose and lipid metabolism, mitochondrial activity, and energy homeostasis. In immune cells, AR signaling influences metabolic reprogramming and polarization through key regulators such as mTOR, AMPK, and HIF-1α, contributing to immune tolerance or activation depending on the context. Additionally, ARs have been implicated in epigenetic modulation, affecting DNA methylation, histone acetylation, and non-coding RNA expression via metabolite-sensitive mechanisms. Therapeutically, AR-targeting agents are being explored for cancer and chronic inflammatory diseases. While clinical trials with A2A antagonists in oncology show encouraging results, challenges remain due to receptor redundancy, systemic effects, and the need for tissue-specific selectivity. Future strategies involve biased agonism, allosteric modulators, and combination therapies guided by biomarker-based patient stratification. Overall, ARs are emerging as integrative hubs connecting extracellular signals with cellular metabolic and epigenetic machinery. Understanding these non-canonical roles may unlock novel therapeutic opportunities across diverse disease landscapes.

## 1. Introduction

**Figure 1:**
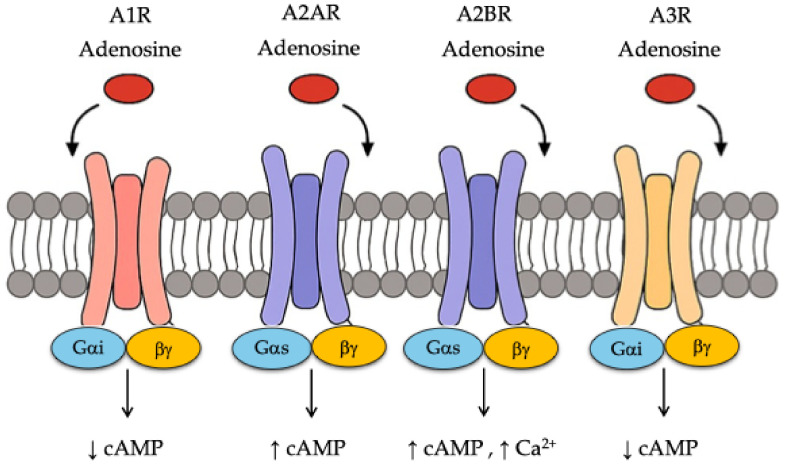
Structure and functioning of adenosine receptors.

**Figure 2:**
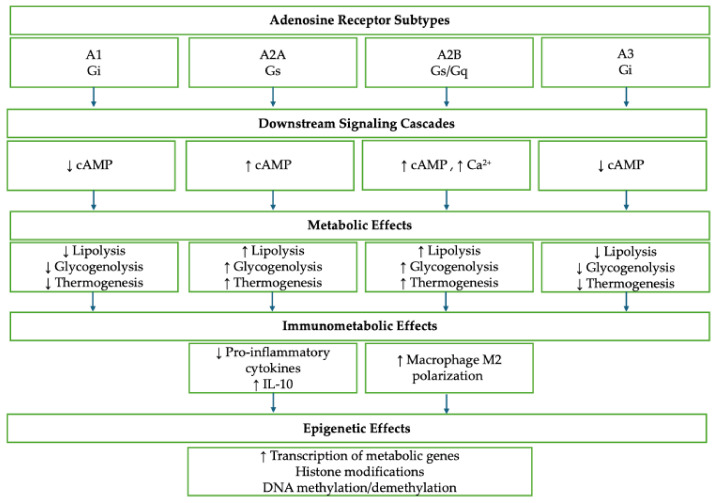
Adenosine receptors downstream signaling cascades and their metabolic, immunometabolic, and epigenetic effects.

Adenosine, a purine nucleoside, is a pivotal signaling molecule that is involved in various physiological and pathological processes, particularly under stress conditions such as hypoxia, inflammation, or ischemia. It exerts its effects through four G protein-coupled receptors (GPCRs), A1, A2A, A2B, and A3, each with distinct affinities and tissue distributions (Figure 1) [1]. Traditionally, these adenosine receptors (ARs) have been studied in the context of cardiovascular function, neuromodulation, and inflammatory regulation, where they control heart rate, vasodilation, neurotransmitter release, and cytokine production [2]. However, recent findings have revealed a broader spectrum of biological activities, highlighting “non-canonical” functions of ARs that extend beyond classical cell signaling. These include the modulation of cellular metabolism, immunometabolic rewiring, and epigenetic regulation, particularly in pathological conditions such as cancer, neurodegeneration, autoimmune disorders, and metabolic diseases (Figure 2) [3,4,5,6,7].

In cancer, the tumor microenvironment (TME) is often enriched in extracellular adenosine due to increased activity of CD39 and CD73 ectonucleotidases. This immunosuppressive milieu reshapes immune cell behavior and metabolism via activation of A2A and A2B receptors, which inhibit cytotoxic responses and promote tolerance [8]. Notably, antagonism of A2B receptors has been shown to disrupt metabolic pathways involved in oxidative stress control and energy production in non-small cell lung cancer (NSCLC) cells, suggesting therapeutic relevance [9].

Immunometabolism—an emerging field at the intersection of metabolism and immune cell function—has further emphasized the centrality of adenosine in regulating macrophage polarization and T cell fate. Activation of A2A receptors in macrophages promotes an M2-like, anti-inflammatory phenotype with reduced cytokine production and increased expression of tissue repair markers [10]. In T cells, A2A signaling restricts proliferation and cytokine release, particularly in chronic inflammation and tumors [11].

In addition to receptor-mediated effects, adenosine influences epigenetic modifications through its metabolism. The nuclear isoform of adenosine kinase (ADK-L) regulates DNA methylation by modulating the availability of S-adenosylmethionine (SAM), a key methyl donor for DNA and histone methyltransferases. Overexpression of ADK-L is linked to epigenetic silencing in cancer and neurodevelopmental disorders [12].

Furthermore, adenosine’s role extends to the central nervous system (CNS), where it regulates various cellular responses, such as insulin sensitivity, lipid metabolism, neuroinflammation, and synaptic function by binding with adenosine receptors. These receptors play distinct roles in processes such as endothelial cell homeostasis, insulin sensitivity, microglial activation, lipid metabolism, immune cell infiltration, and synaptic plasticity. Moreover, recent findings highlight the importance of adenosine signaling in the development of therapeutic solutions for neuropathological issues in patients with metabolic syndromes [13,14].

Therefore, this review aims to synthesize recent advances in understanding the non-canonical roles of adenosine receptors and adenosine metabolism in cellular metabolism, immunometabolism, and epigenetic regulation. A deeper comprehension of these pathways may uncover novel therapeutic opportunities in oncology, neurodegeneration, and immunometabolic diseases.

## 2. Adenosine Receptors and Metabolic Regulation

ARs have emerged as pivotal regulators of cellular metabolism, extending beyond their classical roles in signal transduction. Through modulation of cyclic adenosine monophosphate (cAMP) levels and downstream signaling cascades, ARs influence key metabolic processes such as glucose uptake, lipid oxidation, mitochondrial function, and energy homeostasis [15]. The A1 receptor (A1R), predominantly expressed in adipose tissue, plays a central role in conserving energy under stress conditions. Activation of A1R decreases cAMP levels via Gi protein coupling, leading to inhibition of lipolysis in adipocytes and reduced gluconeogenesis in hepatocytes [16,17]. In adipose tissue, A1R activation has been shown to stimulate leptin secretion, a hormone that is involved in appetite regulation and energy expenditure [18]. Studies using A1R knockout mice have demonstrated increased lipolysis and decreased lipogenesis, indicating the receptor’s role in maintaining lipid balance [19]. Furthermore, pharmacological agents targeting A1R are being explored for their potential to modulate free fatty acid release, offering therapeutic avenues for insulin resistance and hyperlipidemia [16].

Conversely, A2A and A2B receptors (A2AR and A2BR) promote metabolic activation in specific contexts (Table 1). A2BR, with lower affinity for adenosine but higher expression in metabolic organs, is upregulated under hypoxic or inflammatory conditions. In hepatocytes and skeletal muscle, A2BR stimulation enhances glycolytic flux and insulin sensitivity via the AMP-activated protein kinase (AMPK) and phosphoinositide 3-kinase (PI3K)-Akt pathways [20]. A2AR has also been shown to stimulate mitochondrial biogenesis and fatty acid oxidation in cardiac and skeletal muscle cells, highlighting its potential protective role against metabolic stress [21]. In adipose tissue, adenosine functions as a paracrine/autocrine factor that modulates adipocyte differentiation and lipogenesis. Activation of A2BR promotes beige adipocyte formation and enhances energy expenditure through peroxisome proliferator-activated receptor gamma coactivator 1-alpha (PGC-1α) and uncoupling protein 1 (UCP1)-dependent mechanisms [15]. Moreover, A2AR activation has been associated with increased adiponectin secretion, an adipokine that is known for its insulin-sensitizing and anti-inflammatory properties [16]. Furthermore, mitochondrial metabolism is directly influenced by adenosine receptor signaling. Thus, A1R activation has been shown to limit mitochondrial respiration and ATP production in a cell-specific manner, contributing to energy conservation during metabolic stress [22]. On the other hand, A2AR and A2BR can enhance oxidative phosphorylation (OXPHOS) and mitochondrial mass, especially in immune and muscle cells, via upregulation of PGC-1α and nuclear respiratory factors 1 and 2 (NRF1/2) transcription factors [23]. In chondrocytes, A2AR stimulation has been demonstrated to enhance mitochondrial metabolism and mitigate reactive oxygen species (ROS)-mediated mitochondrial injury, suggesting a protective role in maintaining mitochondrial integrity [24].

Disruptions in adenosine signaling have been implicated in metabolic disorders, including obesity, insulin resistance, and type 2 diabetes. For instance, decreased A2AR activity correlates with impaired glucose homeostasis and low-grade inflammation in high-fat-diet-induced obesity models [25]. Additionally, A2BR knockout mice exhibit glucose intolerance and altered lipid profiles, underscoring its role in systemic metabolic regulation. The therapeutic potential of modulating ARs in metabolic disease is gaining recognition. Selective A1R agonists are being evaluated for their glucose-lowering effects via inhibition of hepatic glucose production [16], while A2BR agonists show promise in restoring metabolic balance in insulin-resistant states [26]. Collectively, these findings support a model in which adenosine receptors integrate environmental and cellular stress signals to finely tune metabolic pathways across diverse tissues, offering novel avenues for metabolic disease intervention.

**Table 1:** Metabolic effects of adenosine receptors.

Receptor	Tissue(s)	Signaling Pathways	Metabolic Effects	References
A1R	Adipose, Liver	↓ cAMP (via Gi), Leptin signaling	↓ Lipolysis ↓ Gluconeogenesis ↑ Leptin secretion ↓ ATP production	[16,17]
A2AR	Heart, Muscle, Immune Cells	AMPK, PI3K-Akt, PGC-1α, NRF1/2	↑ FA oxidation ↑ Mitochondrial biogenesis ↑ Adiponectin Protection from ROS injury	[16,21,23,24]
A2BR	Liver, Muscle, Adipose	AMPK, PI3K-Akt, PGC-1α, UCP1	↑ Glycolysis ↑ Insulin sensitivity ↑ Beige adipocytes ↑ Energy expenditure	[15,20,23]
Combined	Systemic	Various	Integration of stress signals Metabolic adaptation Potential therapeutic targets	[16,26]

## 3. Immunometabolic Functions of Adenosine Receptors

Immunometabolism is an emerging field that explores the bidirectional relationship between immune cell function and cellular metabolism. Within the tumor microenvironment (TME), chronic inflammation, hypoxia, and nutrient depletion trigger metabolic adaptations that modulate immune responses, often tipping the balance toward immune suppression and tumor progression [27]. Adenosine, a key metabolite that accumulates under hypoxic and inflammatory conditions, plays a pivotal immunomodulatory role through its interaction with four G protein-coupled adenosine receptors (A1R, A2AR, A2BR, and A3R) [28].

In the TME, extracellular adenosine is generated through the sequential hydrolysis of ATP by the ectonucleotidases CD39 and CD73, which are frequently upregulated in hypoxic tumors [29]. Hypoxia-inducible factor-1α (HIF-1α) enhances CD73 expression, further promoting adenosine accumulation [11]. Elevated adenosine concentrations (>100 µM) are typical in solid tumors and correlate with increased immunosuppressive activity [30]. Among the adenosine receptors, A2AR and A2BR are the most implicated in modulating immune responses. A2AR is abundantly expressed on activated CD8+ T cells, and its stimulation suppresses proliferation, granzyme B expression, and IFN-γ release, thereby dampening cytotoxic function [31]. A2BR is highly expressed on myeloid cells, including macrophages and dendritic cells, where it promotes anti-inflammatory cytokine secretion (e.g., IL-10, TGF-β) and inhibits antigen presentation [32].

In T regulatory cells (Tregs), adenosine signaling via A2AR enhances the suppressive capacity and FOXP3 expression, reinforcing the immunosuppressive tone of the TME [33]. Natural killer (NK) cells are similarly affected; A2AR activation reduces their cytotoxicity and expression of perforin and IFN-γ [11]. Adenosine can also influence macrophage polarization. It promotes the M2-like phenotype associated with tissue repair and tumor progression and inhibits M1-like proinflammatory responses [34]. In dendritic cells, adenosine impairs maturation and reduces the expression of co-stimulatory molecules (CD80, CD86), thereby limiting effective T cell priming [35].

Given the central role of adenosine in immune evasion, several therapeutic strategies aim to interfere with its signaling. These include inhibitors of CD39 and CD73 (to prevent adenosine production) and antagonists of A2AR and A2BR (to block receptor-mediated effects) [35]. A2AR antagonists such as ciforadenant and AZD4635 have shown promise in early-phase clinical trials, particularly when combined with immune checkpoint inhibitors [36]. Preclinical studies indicate that blockade of the adenosinergic axis restores effector functions of CD8+ T cells and NK cells, reprograms tumor-associated macrophages, and improves the efficacy of PD-1/PD-L1 and CTLA-4 blockade therapies [37,38]. Dual blockade of A2AR and CD73 shows synergistic effects in models of melanoma, breast, and colorectal cancer [39]. Moreover, the adenosinergic pathway is being explored beyond oncology (Table 2). In chronic infections and autoimmunity, adenosine plays dual roles, either restraining inflammation or promoting persistence of pathogens and immune exhaustion [40].

**Table 2:** Immunosuppressive role of adenosine in the tumor microenvironment (TME).

Process/Component	Description/Effect	References
Adenosine production pathway	ATP → AMP (via CD39) → Adenosine (via CD73); both enzymes upregulated in hypoxic tumors	[11]
Hypoxia and inflammation	Induce HIF-1α expression → upregulation of CD73 → increased extracellular adenosine	[11,29]
Adenosine concentration	>100 µM in solid tumors; correlates with immunosuppression	[30]
A2AR (adenosine A2A receptor)	Expressed on CD8^+^ T cells, Tregs, NK cells; suppresses IFN-γ, granzyme B, perforin; enhances FOXP3	[31]
A2BR (adenosine A2B receptor)	Expressed on macrophages and dendritic cells; promotes IL-10, TGF-β; inhibits antigen presentation	[32]
Tregs (regulatory T cells)	A2AR activation enhances suppressive capacity and FOXP3 expression	[33]
Macrophage polarization	Adenosine promotes M2 phenotype (pro-tumor) and inhibits M1 phenotype (proinflammatory)	[34]
Dendritic cells (DCs)	Adenosine impairs maturation and co-stimulatory molecule expression (CD80, CD86)	[35]
Therapeutic strategies	-CD39/CD73 inhibitors reduce adenosine production—A2AR/A2BR antagonists (e.g., ciforadenant, AZD4635)—combination with immune checkpoint inhibitors (PD-1, CTLA-4)	[36,37,38,39]
Beyond oncology	Adenosine modulates immune responses in chronic infections and autoimmunity (dual pro-/anti-inflammatory role)	[40]

## 4. Epigenetic Regulation via Adenosine Signaling

Epigenetic modifications, encompassing DNA methylation, histone modifications, chromatin remodeling, and non-coding RNAs, play a pivotal role in regulating gene expression without altering the underlying DNA sequence. While other omics approaches such as proteomics, transcriptomics, and metabolomics have significantly advanced our understanding of adenosine-related pathways, the epigenetic dimension remains comparatively underexplored.

In particular, in the context of cancer, these modifications contribute significantly to tumor progression and immune evasion by reprogramming both tumor cells and the surrounding TME [41]. Within the TME, epigenetic dysregulation affects not only cancer cells but also various immune cell populations. For instance, tumor-associated macrophages (TAMs) and myeloid-derived suppressor cells (MDSCs) can acquire immunosuppressive phenotypes through epigenetic mechanisms, thereby facilitating tumor growth and metastasis [42]. Moreover, regulatory T cells (Tregs) exhibit enhanced suppressive functions due to epigenetic changes that stabilize their lineage and function [43].

Hypoxic conditions, which are prevalent in solid tumors, further exacerbate epigenetic alterations. Hypoxia-inducible factors (HIFs) can modulate the expression of various epigenetic enzymes, leading to widespread changes in gene expression that promote angiogenesis, metabolic reprogramming, and immune suppression [8]. Epigenetic mechanisms also regulate the expression of immune checkpoint molecules such as PD-L1 and CTLA-4. DNA methylation and histone modifications can upregulate these checkpoints on tumor cells, thereby inhibiting effective T cell responses [44]. Additionally, the epigenetic silencing of genes involved in antigen processing and presentation impairs the ability of tumor cells to be recognized by cytotoxic T lymphocytes, facilitating immune escape.

Recent findings reveal that adenosine metabolism, particularly through the activity of CD39 and CD73, influences epigenetic regulation by modulating intracellular levels of S-adenosylmethionine (SAM) and α-ketoglutarate—critical cofactors for DNA and histone methyltransferases and demethylases. Elevated extracellular adenosine can alter the balance of these metabolites in immune and tumor cells, indirectly affecting DNA methylation and histone modifications [45,46]. For example, adenosine A2A receptor (A2AR) signaling has been shown to suppress TET enzyme activity and reduce DNA demethylation in T cells, contributing to their functional exhaustion and impaired memory differentiation [25]. Moreover, adenosine-rich environments can modulate the expression of chromatin remodelers such as EZH2 and HDAC1 via cAMP-PKA signaling cascades downstream of A2AR and A2BR activation, thereby reinforcing an immunosuppressive transcriptional program in the TME [47]. Non-coding RNAs, including microRNAs (miRNAs) and long non-coding RNAs (lncRNAs), are also influenced by adenosine signaling. A2BR activation in cancer-associated fibroblasts has been linked to the upregulation of miR-146a and lncRNA NEAT1, both of which are known to dampen immune activation and promote a pro-tumoral phenotype [48]. Conversely, inhibition of adenosine signaling can restore the expression of tumor-suppressive miRNAs, such as miR-34a and let-7 family members, with downstream effects on cell cycle arrest and immune surveillance.

Given the reversible nature of epigenetic modifications, targeting epigenetic enzymes presents a promising therapeutic strategy. Inhibitors of DNA methyltransferases (DNMTs) and histone deacetylases (HDACs) have shown potential in reactivating silenced tumor suppressor genes and enhancing tumor immunogenicity. Moreover, combining epigenetic therapies with immune checkpoint inhibitors has demonstrated synergistic effects in preclinical models, leading to improved antitumor immune responses (Table 3). Recent clinical trials have explored the efficacy of such combination therapies. For example, the use of DNMT inhibitors in conjunction with PD-1 blockade has shown enhanced therapeutic outcomes in certain cancer types. Furthermore, targeting specific epigenetic regulators like EZH2 and BET proteins is under investigation for their roles in modulating the TME and overcoming resistance to immunotherapy [27].

Notably, epigenetic drugs such as azacytidine (a DNMT inhibitor) and entinostat (a class I HDAC inhibitor) have been shown to modulate adenosine signaling pathways. Azacytidine reduces CD73 expression in AML and MDS models, potentially decreasing adenosine accumulation and enhancing antitumor immunity [49]. Entinostat has been reported to suppress A2AR expression on myeloid cells and Tregs, thereby improving T cell responsiveness when combined with anti-PD-1 therapy [29]. These findings support the rationale for combining epigenetic modulators with adenosine receptor antagonists to simultaneously reprogram the epigenome and relieve metabolic immunosuppression in the TME.

## 5. Targeting Adenosine Receptors in Cancer Therapy

The growing understanding of non-canonical functions of adenosine receptors in metabolism, immunometabolism, and epigenetic regulation has opened novel avenues for therapeutic interventions in various human diseases, especially cancer and chronic inflammatory disorders. Adenosine accumulates in TME as a result of hypoxia-induced ATP release and subsequent degradation by ectonucleotidases CD39 and CD73. This leads to activation of ARs, particularly the high-affinity A2AR, which plays a key role in suppressing the immune response and promoting tumor immune escape [50]. Activation of A2AR inhibits the cytotoxic functions of CD8+ T cells and NK cells, reduces the proliferation of helper T cells, and promotes the expansion of Tregs, thus creating a profoundly immunosuppressive milieu [27]. Pharmacologic inhibition of A2AR has shown considerable promise in preclinical cancer models. A2AR antagonists restore effector T cell activity, enhance IFN-γ production, and increase tumor infiltration by immune cells, resulting in slowed tumor growth or even regression [47]. This has led to the clinical development of selective A2AR antagonists such as ciforadenant (CPI-444), etrumadenant (AB928), and AZD4635, which are currently being tested in clinical trials as monotherapy or in combination with checkpoint inhibitors [51,52].

However, several A2AR antagonists have failed to meet clinical endpoints in early-phase trials due to multiple factors. First, despite robust preclinical efficacy, many compounds such as ciforadenant showed only limited clinical benefits when used as monotherapy, likely due to insufficient immune activation in patients with a low adenosine burden or poor tumor infiltration by effector lymphocytes [53]. Second, dose-limiting toxicities and off-target effects—particularly central nervous system-related adverse events given the widespread expression of A2AR in the brain—have constrained the optimal dosing and therapeutic window [29]. Third, trial designs often lacked adequate patient stratification based on tumor adenosinergic signatures (e.g., CD73 expression or A2AR PET imaging), potentially diluting efficacy signals. Moreover, compensatory upregulation of alternative immunosuppressive pathways (e.g., A2BR, PD-L1, IDO1) may limit the benefit of single-agent A2AR blockade, suggesting the need for rational combination strategies [29,51]. These clinical observations underscore the necessity of integrating biomarker-guided selection, combination regimens, and improved pharmacokinetic profiles in the next generation of adenosine-targeting therapies.

Emerging evidence also suggests a functional role for the low-affinity A2BR, particularly in myeloid cells such as TAMs, dendritic cells, and MDSCs. A2BR signaling promotes a pro-tumoral phenotype by inducing VEGF production, impairing antigen presentation, and supporting immunosuppressive cytokine release (e.g., IL-10, TGF-β) [54]. Dual A2AR/A2BR blockade is therefore being explored as a potentially more effective strategy to reprogram the immune TME, particularly in tumors with a high adenosine burden or low responsiveness to anti-PD-1/PD-L1 monotherapies [37]. Additionally, CD73, the enzyme that is responsible for AMP-to-adenosine conversion, is now recognized as a critical upstream regulator of adenosine signaling. High CD73 expression in several tumor types (e.g., triple-negative breast cancer, ovarian, and colorectal cancer) correlates with poor prognosis and immune resistance. Therapeutic antibodies targeting CD73 (e.g., oleclumab) are under active investigation in combination regimens to reduce adenosine accumulation and enhance the efficacy of AR antagonists and ICIs [55,56].

Importantly, the integration of AR blockade into broader immunotherapy protocols has led to the concept of “metabolic checkpoint blockade,” where metabolic pathways like adenosine signaling are targeted alongside classical immune checkpoints. Preclinical studies demonstrate that co-inhibition of A2AR and PD-1 significantly boosts T cell metabolic fitness, reverses T cell exhaustion, and increases the tumor-killing capacity of adoptively transferred T cells in murine cancer models [2]. Personalized approaches, based on the tumor adenosinergic signature, receptor expression profiling, and functional imaging (e.g., PET tracers for CD73 or A2AR), may further refine the therapeutic index of adenosine-targeted therapies in the clinical setting [57].

## 6. Targeting Adenosine Receptors in Chronic Inflammatory Disorders

Adenosine signaling plays a pivotal role in the modulation of immune responses and the resolution of inflammation. Its concentration increases markedly during tissue injury, hypoxia, and metabolic stress, making it a critical regulatory signal in chronic inflammatory disorders such as rheumatoid arthritis (RA), inflammatory bowel disease (IBD), psoriasis, chronic obstructive pulmonary disease (COPD), and systemic lupus erythematosus (SLE) [58]. Adenosine exerts its effects through four G protein-coupled receptors (A1, A2A, A2B, A3), each exhibiting distinct tissue distributions and immunomodulatory functions. The A2A receptor (A2AR) is generally associated with anti-inflammatory effects. In RA, A2AR activation on monocytes, macrophages, and effector T cells reduces the secretion of proinflammatory cytokines such as TNF-α, IL-6, and IL-1β, while promoting IL-10 release and suppressing matrix metalloproteinase expression [59]. The clinical relevance of A2AR is exemplified by methotrexate, whose anti-inflammatory mechanism partly relies on extracellular adenosine accumulation and A2AR activation [60]. More recently, preclinical studies using A2AR agonists, including CGS21680 and regadenoson, have shown promise in attenuating synovial inflammation and bone erosion in experimental arthritis models [61].

The A3 receptor (A3R) has also gained attention in chronic inflammation due to its expression on neutrophils, mast cells, and fibroblasts. A3R agonists, such as IB-MECA (CF101), have demonstrated efficacy in phase II clinical trials for RA and psoriasis by reducing leukocyte infiltration and modulating the PI3K/Akt and NF-κB pathways [9]. Moreover, the ability of A3R ligands to selectively accumulate in inflamed tissues confers a therapeutic advantage by limiting systemic immunosuppression [62]. In contrast, the A2B receptor (A2BR) displays dualistic effects. While its activation can promote epithelial barrier repair and mucus secretion in the early inflammatory stages of IBD, it also enhances the release of IL-6 and IL-8 from macrophages and stromal cells, thereby perpetuating chronic inflammation and fibrosis [63]. For example, Kolachala et al. showed that A2BR activation promotes intestinal epithelial restitution and barrier integrity in acute injury models [64], whereas Frick et al. reported that sustained A2BR signaling drives proinflammatory cytokine production and fibrosis in chronic colitis [65]. Similarly, in the lung, A2BR stimulation has been shown to induce pro-fibrotic gene expression in fibroblasts and increase TGF-β production, contributing to pulmonary fibrosis [66], although other reports have suggested that A2BR agonism might also suppress neutrophil recruitment under specific conditions [67]. These opposing findings have led to debates regarding the therapeutic utility of A2BR antagonists versus agonists depending on the disease stage and cellular context. A2BR antagonists such as PSB603 have been shown to ameliorate colitis in murine models by reducing cytokine burden and preserving intestinal architecture [68].

In skin inflammation, particularly in psoriatic disease, adenosine receptor modulation has yielded encouraging results. A2AR agonists downregulate Th17-related cytokines (IL-17A, IL-22), inhibit keratinocyte hyperproliferation, and reduce neutrophilic infiltration, suggesting a potential role as topical or systemic adjuncts to biologic therapies [69]. A3R ligands are also being evaluated for their antiproliferative effects on keratinocytes and regulatory actions on dermal immune cells [70]. In respiratory diseases such as COPD and asthma, A2BR expression in bronchial epithelial cells, mast cells, and fibroblasts contributes to airway remodeling, collagen deposition, and recruitment of neutrophils and eosinophils [71]. Inhibition of A2BR reduces airway hyperresponsiveness, mucus overproduction, and inflammatory cytokine release, highlighting its potential as a target for inhaled therapy in chronic airway diseases [66].

Importantly, dual blockade of A2AR and A2BR has been proposed as a novel anti-fibrotic strategy, particularly in fibrotic forms of IBD and pulmonary inflammation, where both receptor subtypes are upregulated in activated myofibroblasts and immune infiltrates [72]. Emerging interest has also turned to the modulation of adenosine metabolism upstream of receptor activation. Inhibition of CD39 and CD73, the enzymes that are responsible for the stepwise conversion of extracellular ATP to adenosine, is under evaluation as a strategy to limit excessive adenosine production in chronic inflammation. Selective CD73 inhibitors, such as APCP and MEDI9447, have shown efficacy in attenuating inflammation and fibrosis in murine models of colitis and liver injury [73].

Together, these findings underscore the therapeutic potential of selectively modulating adenosine receptors in a tissue- and disease-specific manner (Table 4). The development of next-generation AR ligands, with improved pharmacokinetics and reduced off-target effects, coupled with local delivery systems and patient stratification strategies, holds promise for effective and safe management of chronic inflammatory conditions.

## 7. Challenges and Future Directions

Despite the growing body of evidence supporting the therapeutic potential of adenosine receptors (ARs) in metabolic, inflammatory, and epigenetic contexts, several critical challenges remain that hinder the translation of preclinical findings into clinical success. One major obstacle is the ubiquitous expression and context-dependent activity of AR subtypes, which often results in complex and sometimes contradictory effects across tissues and disease models [74]. For instance, while A2A receptor activation generally exerts anti-inflammatory and immunosuppressive effects, its chronic stimulation may impair host defense mechanisms or promote immunosuppression in tumor microenvironments [38]. Similarly, A2B receptors have been implicated both in tissue repair and in fibrogenesis, depending on the timing and cellular context [63].

Another major limitation lies in the pharmacological tools that are currently available. Many AR agonists and antagonists lack sufficient receptor subtype selectivity and exhibit suboptimal pharmacokinetics, leading to off-target effects or poor tissue penetration [75]. Moreover, long-term use of AR ligands may induce receptor desensitization or tolerance, further complicating their therapeutic application [29]. The development of biased ligands or allosteric modulators—capable of fine-tuning receptor signaling without triggering global activation—represents a promising but still underexplored avenue [76]. A further layer of complexity is added by the dynamic regulation of extracellular adenosine levels through the CD39–CD73 enzymatic axis and adenosine deaminase activity, which are themselves influenced by hypoxia, inflammation, and cellular metabolism [77].

Targeting adenosine production and degradation in parallel with AR modulation may offer synergistic benefits, but this requires a more refined understanding of disease-specific adenosine dynamics. From a clinical perspective, a major unmet need is the identification of predictive biomarkers for patient stratification. Given the heterogeneous expression of ARs in different tissues and pathologies, companion diagnostics assessing receptor density, adenosine levels, or expression of regulatory enzymes may enhance therapeutic precision and minimize adverse effects [78]. Additionally, the integration of adenosinergic therapies with existing immunotherapies, anti-inflammatory agents, or metabolic modulators requires rigorous investigation to define optimal combinations and dosing strategies. Future research should also prioritize the elucidation of AR functions in human tissues using single-cell transcriptomic and spatial proteomic approaches. These technologies can uncover cell-type-specific receptor expression patterns and context-dependent signaling cascades, especially within complex microenvironments such as tumors, fibrotic niches, and inflamed mucosal tissues [79].

Furthermore, the emerging roles of ARs in epigenetic regulation, microbiome–host interactions, and neuroimmune crosstalk represent fertile grounds for discovery with potential systemic implications. In summary, advancing adenosine receptor research from bench to bedside will require a concerted effort to overcome current pharmacological, biological, and clinical limitations. By embracing multidisciplinary approaches and precision medicine frameworks, the field is poised to unlock the full therapeutic potential of adenosinergic signaling across a broad spectrum of human diseases.

## 8. Conclusions

In recent years, the landscape of AR research has significantly evolved, moving beyond traditional views of ARs as mere modulators of cardiovascular tone and neurotransmission. Emerging evidence underscores their critical roles in diverse cellular processes, including metabolism, immunometabolism, and epigenetic regulation, positioning ARs as multifaceted players in health and disease. The intricate interplay between AR subtypes and intracellular signaling networks confers upon them the ability to fine-tune cellular responses to metabolic stress, inflammation, and environmental cues.

This review highlights the non-canonical functions of ARs in shaping metabolic homeostasis, modulating immune cell energetics, and influencing epigenetic landscapes, with particular emphasis on their implications for chronic inflammatory conditions and cancer.

Moreover, emerging evidence suggests that adenosine receptors may play a significant role in inflammaging, the chronic, low-grade inflammation that accumulates with age and contributes to age-related diseases. In particular, the A2A and A2B receptors have been shown to exert anti-inflammatory effects by modulating the activity of macrophages, T cells, and other immune cells that are commonly involved in inflammaging processes. Dysregulation of AR signaling in aging tissues may impair the resolution of inflammation, alter immune cell metabolism, and contribute to tissue degeneration. Given these observations, targeting ARs could represent a novel strategy to counteract inflammaging and its downstream pathological consequences. Future studies should further investigate the age-dependent expression and function of ARs in various tissues, as well as their therapeutic potential in delaying or mitigating age-related inflammatory disorders [80,81,82].

The therapeutic promise of targeting adenosine signaling is underscored by the clinical development of AR ligands and inhibitors of the adenosine-generating ectoenzymes CD39 and CD73. Nonetheless, the widespread distribution of ARs and the complexity of their context-dependent functions present significant challenges for drug development and clinical translation.

A deeper understanding of ARs’ biology will require the integration of advanced experimental strategies. These include single-cell transcriptomic and proteomic analyses to resolve cell-type-specific AR expression patterns, the development of conditional knockout or reporter mouse models to study receptor function in vivo, and high-resolution imaging techniques to track AR activation and signaling dynamics in real time. Additionally, combining functional assays with metabolic and epigenetic profiling could reveal novel roles of ARs in specific disease contexts. Such approaches will be essential for translating basic insights into targeted therapeutic applications [83,84,85,86].

Furthermore, personalized medicine strategies that incorporate biomarker-guided patient selection, tissue-specific delivery systems, and combinatorial regimens with immunomodulatory or metabolic agents may be essential for maximizing the therapeutic potential of AR-targeted interventions.

Altogether, adenosine receptors represent a dynamic and promising therapeutic axis. Bridging fundamental discoveries with translational insights will be key to harnessing their full clinical utility across a wide spectrum of metabolic, immune-mediated, and epigenetically regulated diseases.

## Figures and Tables

**Table 3:** Epigenetic mechanisms in cancer: impact on tumor progression, immune evasion, and therapeutic opportunities.

Epigenetic Mechanism	Effects in the Tumor Microenvironment (TME)	Impacted Cell Types/Functions	Therapeutic Implications	References
DNA methylation	Silencing of tumor suppressor genes; upregulation of immune checkpoints (e.g., PD-L1, CTLA-4); inhibition of antigen presentation	Tumor cells; cytotoxic T cells (via impaired recognition)	DNMT inhibitors restore gene expression and immunogenicity	[44]
Histone modifications	Altered chromatin structure; regulation of immune-related genes; stabilization of Treg phenotype	Tregs; tumor cells	HDAC inhibitors used to reverse suppressive histone marks	[45,46]
Chromatin remodeling	Affects accessibility of immune genes and checkpoint molecules; supports tumor-promoting transcriptional programs	TAMs, MDSCs, tumor cells	Targeting specific remodeling enzymes (e.g., EZH2) in clinical testing	[47]
Non-coding RNAs	Post-transcriptional regulation of immune modulators and oncogenes	Multiple immune cells and tumor cells	miRNA and lncRNA-based therapies under investigation	[48]
Hypoxia-induced epigenetic changes	HIFs regulate epigenetic enzymes → promote angiogenesis, immune suppression, metabolic reprogramming	Tumor cells, immune cells	Indirect targeting via hypoxia modulation and HIF pathway inhibitors	[8]
Effect on immune cells	-TAMs/MDSCs: epigenetically driven immunosuppressive phenotypes; Tregs: epigenetically stabilized suppressive function	TAMs, MDSCs, Tregs	Modulation of epigenetic pathways may reprogram immune cells	[42,43]
Checkpoint regulation	Epigenetic upregulation of PD-L1, CTLA-4 blocks T cell-mediated killing	Tumor cells, T cells	Combos with anti-PD-1/PD-L1 or CTLA-4 + epigenetic drugs	[27,44]
Clinical strategies	Use of DNMT and HDAC inhibitors to enhance tumor immunogenicity; combination with ICI therapies showing synergistic effects	Tumor and immune compartments	Clinical trials testing DNMTi/HDACi + checkpoint inhibitors	[49]
Emerging targets	EZH2, BET proteins, and others being explored for immunomodulatory roles	Tumor cells, TME components	Potential to overcome resistance to immunotherapy	[27,47]

**Table 4:** Disease-specific roles of adenosine receptors (ARs): tissue targets, signaling pathways, and therapeutic interventions.

Disease	Organ/Tissue/ Cells Affected	AR Involved	Signaling Pathway/ Effect	Drug/Intervention	References
Obesity/Insulin Resistance	Adipose tissue, hepatocytes	A1R	↓ cAMP → ↓ lipolysis, ↓ gluconeogenesis; ↑ leptin secretion	N^6-cyclopentyladenosine (CPA), GR79236	[16]
Type 2 Diabetes	Liver, skeletal muscle	A2BR	↑ AMPK, ↑ PI3K/Akt → ↑ glycolysis, ↑ insulin sensitivity	BAY 60-6583	[20,26]
Metabolic Syndrome	Adipocytes	A2AR, A2BR	↑ Adiponectin secretion; ↑ PGC-1α/UCP1 → beige adipocyte formation	CGS21680, BAY 60-6583	[15,16]
Cardiac/Skeletal Myopathy	Cardiomyocytes, muscle fibers	A2AR	↑ Mitochondrial biogenesis, ↑ fatty acid oxidation via PGC-1α, NRF1/2	CGS21680, ATL146e	[21,23]
Osteoarthritis/Cartilage Damage	Chondrocytes	A2AR	↑ Mitochondrial metabolism; ↓ ROS-mediated mitochondrial injury	CGS21680, ATL313	[24]
Cancer (Solid Tumors)	TME, CD8^+^ T cells, Tregs, NK cells, macrophages	A2AR, A2BR	↑ FOXP3, ↓ IFN-γ, ↓ granzyme B, ↑ IL-10/TGF-β, ↑ M2 macrophages	Ciforadenant (CPI-444), AZD4635, Oleclumab (MEDI9447), AB928	[28,39,47,49]
Rheumatoid Arthritis (RA)	Synovium, monocytes, T cells, fibroblasts	A2AR, A3R	↓ TNF-α, IL-6, IL-1β; ↑ IL-10; modulates PI3K/Akt, NF-κB	Methotrexate, CGS21680, IB-MECA (CF101)	[59]
Psoriasis	Skin, keratinocytes, Th17 cells	A2AR, A3R	↓ IL-17A, IL-22; ↓ keratinocyte proliferation; ↓ neutrophil infiltration	Piclidenoson (CF101)	[69,70]
IBD (Ulcerative Colitis, Crohn’s)	Gut epithelium, macrophages, stromal cells	A2BR	↑ Barrier integrity (acute); ↑ IL-6, IL-8, fibrosis (chronic)	PSB603, CVT-6883	[63,65,68]
COPD/Asthma	Airway epithelium, fibroblasts, mast cells	A2BR	↑ TGF-β, collagen; airway remodeling, neutrophil recruitment	GS-6201, CVT-6883	[66,71]
Liver Fibrosis/Inflammation	Hepatic stellate cells, fibroblasts	A2AR, A2BR	↑ Pro-fibrotic cytokines; ↑ collagen synthesis	AB928 (etrumadenant)	[72,73]
Autoimmunity (SLE, MS)	Tregs, Th cells, myeloid cells	A2AR, A3R	↑ FOXP3 expression; ↓ effector T cell activation	Piclidenoson (CF101), Namodenoson (CF102)	[58,62]
Chronic Infections	T cells, macrophages	A2AR, A2BR	Promotes T cell exhaustion, pathogen persistence	Ciforadenant, AZD4635	[40]
